# The Development of Standardized National Head Circumference Growth Charts for Jordanian Children Aged 0–5 Years: A Longitudinal and Cross-Sectional Study

**DOI:** 10.3390/children12020224

**Published:** 2025-02-13

**Authors:** Walid Al-Qerem, Anan Jarab, Ahmad Al-Azayzih, Judith Eberhardt, Ruba Zumot, Fawaz Alasmari, Alaa Hammad

**Affiliations:** 1 Department of Pharmacy, Faculty of Pharmacy, Al-Zaytoonah University of Jordan, Amman 11733, Jordan; waleed.qirim@zuj.edu.jo (W.A.-Q.); 202127091@std-zuj.edu.jo (R.Z.); alaa.hammad@zuj.edu.jo (A.H.); 2 College of Pharmacy, Al Ain University, Abu Dhabi 112612, United Arab Emirates; anan.jarab@aau.ac.ae; 3 Department of Clinical Pharmacy, Faculty of Pharmacy, Jordan University of Science and Technology, Irbid 22110, Jordan; aaazayzih@just.edu.jo; 4 Department of Pharmacy Practice and Pharmacotherapeutics, College of Pharmacy, University of Sharjah, Sharjah P.O. Box 27272, United Arab Emirates; 5 Department of Psychology, School of Social Sciences, Humanities and Law, Teesside University, Borough Road, Middlesbrough TS1 3BX, UK; 6 Department of Pharmacology and Toxicology, College of Pharmacy, King Saud University, Riyadh 12372, Saudi Arabia; ffalasmari@ksu.edu.sa

**Keywords:** head circumference, growth charts, Jordanian children, pediatric growth patterns, microcephaly, macrocephaly, WHO standards, Lambda–Mu–Sigma statistical method, anthropometric measurements, region-specific growth standards

## Abstract

**Background:** Head circumference (HC) is a key indicator of growth and brain development in children, used to identify abnormalities like microcephaly and macrocephaly. While WHO growth standards are widely adopted, they may not account for regional variations due to genetic, environmental, and socio-economic factors. This study aimed to develop and validate national HC growth charts for Jordanian children aged 0–5 years and compare them with WHO standards. **Method:** This study analyzed 628,456 HC measurements from 250,276 Jordanian children (51.6% boys, 48.4% girls) aged 0–1800 days, using data from the Hakeem program. Only healthy children were included. HC measurements followed international guidelines, and data were analyzed using the Lambda–Mu–Sigma (LMS) statistical method. Percentiles were calculated, and growth patterns were compared with WHO standards. **Results:** National HC-for-age growth charts were developed for the 3rd, 15th, 50th, 85th, and 97th percentiles. Median percentiles for Jordanian children aligned with WHO standards, but the 3rd percentile was lower, particularly for girls after 840 days. The 97th percentile diverged after 240 days, with larger HC measurements observed in Jordanian children. **Conclusions:** This study provides the first validated HC growth charts for Jordanian children, addressing the limitations of international standards in reflecting regional variations. These charts offer clinicians and public health professionals a precise tool for assessing and monitoring growth, promoting better health outcomes in Jordanian children.

## 1. Introduction

Understanding children’s growth and development during the early stages of life is important essential for ensuring positive and healthy developmental outcomes. Head circumference (HC) is widely recognized as a sensitive indicator of children’s growth and brain development, showing a strong correlation with cognitive function during infancy and early childhood [1,2,3]. Examining HC is crucial for screening abnormalities in cranium development, such as macrocephaly and microcephaly, which are often associated with neural dysfunction and developmental delay [4,5]. Macrocephaly is defined as an HC in an infant that is above two standard deviations or greater than the 97th percentile of the population’s average for age and sex. It may result from underlying serious health conditions such as brain tumors, increased intracranial pressure, or increased cerebrospinal fluid. Additionally, macrocephaly can stem from genetic disorders (e.g., Fragile X syndrome), or be benign and familial, where other family members also have a large HC [6]. Microcephaly is characterized by an HC below two standard deviations or less than the 3rd percentile of the reference population for age and sex [7]. Microcephaly may also arise from genetic disorders, prenatal infections (e.g., toxoplasmosis or Zika virus), or prenatal malnourishment [8,9]. Growth charts are widely used as references to evaluate infants’ growth and development, facilitating the early detection and prevention of underlying diseases [10]. Comparing a child’s anthropometric measurements, including HC, to peers of the same age and sex using standardized growth charts serves as a quantitative measure to ensure healthy growth and to determine whether the child is developing normally relative to average standards [11].

The American Academy of Pediatrics and the Child Health and Disability Prevention (CHDP) Program Health Assessment Guidelines (guideline #4) recommend measuring head circumference (HC) during each medical appointment in infancy and early childhood. These measures are intended to ensure healthy growth and to identify potential underlying medical problems [12]. Specifically, The American Academy of Pediatrics advises measuring HC eight times during the first two years of life [13]. The CHDP Program Health Assessment Guidelines also outline the steps involved for measuring HC in newborns and toddlers under the age of two years. It is important to note that differences in a child’s body and growth patterns across countries and regions are influenced by genetic and environmental factors, as well as their interactions [14]. Postpartum HC growth can be affected by various factors, including toxin exposure during pregnancy, prenatal growth restrictions, gestational age, birth weight, nutrition, and socioeconomic status [15,16,17,18,19,20]. Furthermore, both parents’ HC measurements account for approximately half of the variability in HC [21,22]. As a result, accurately identifying deviations from normal HC growth is both crucial and challenging.

To obtain universal HC standards that reflect the diverse backgrounds and environments of healthy infants, the 2000 Centers for Disease Control and Prevention (CDC) HC charts were developed using data from the National Health and Nutrition Examination Survey (NHANES), collected between 1963 and 1994. This dataset comprised five cross-sectional surveys evaluating the HC-for-age of US infants from birth to 36 months by sex [23,24]. Additionally, the World Health Organization (WHO) HC-for-age standards were developed through the Multicenter Growth Reference Study Group (MGRS), which collected data from healthy children under five years, categorized into three age groups by sex: birth to 13 weeks, birth to 2 years, and birth to 5 years [11]. The data were gathered from six countries over six years (1997–2003): the US, Ghana, Oman, Norway, Brazil, and India [25]. Both standards highlight important growth patterns and ethnic diversity and have been applied worldwide [24,25].

The WHO recommends that breastfeeding serves as the standard infant feeding method. Accordingly, the WHO HC charts represent the growth patterns of infants who were breastfed predominantly for at least four months and continued breastfeeding for one year, serving as the standard for growth. Healthcare providers often use CDC HC charts to track the development of older infants. CDC charts are used as a reference to characterize the typical growth and development of children within the United States. WHO growth charts, on the other hand, are designed to indicate how infants should develop under ideal conditions [11,24].

Several countries and healthcare providers, particularly in developed and advanced economies such as China, Germany, Saudi Arabia, and the United Kingdom (UK), use national growth references [26,27]. For instance, the UK has developed nation-specific growth charts that account for the population’s socio-economic and genetic characteristics. The UK’s national anthropometric data were collected and integrated with the WHO’s charts, resulting in UK–WHO growth charts which harmonize with, and more accurately reflect, the growth patterns of the British population [28,29,30].

Despite significant improvements in health and medical services in Jordan, controversies persist regarding the monitoring and evaluation of the growth patterns of Jordanian infants. Studies assessing the validity of international growth standards, their applicability to Jordanian infants, and the necessity of developing Jordanian-specific growth charts to more accurately reflect the growth patterns of infants in this region are limited. This study therefore aimed to address these gaps by developing and validating national head circumference growth charts for Jordanian children aged 0–5 years. Additionally, it sought to evaluate how well international growth standards align with the growth patterns of Jordanian infants.

## 2. Materials and Methods

Across all regions of the kingdom, HC data were collected using both cross-sectional and longitudinal methods from Jordanian infants and children aged 0–5 years. The longitudinal data refers to data collected from the same children in multiple time-points, whereas the cross-sectional data include data of children that had a single measurement. The data collection was conducted through the Hakeem program, a national electronic system that includes medical records for Jordanians registered with the Ministry of Health and the Royal Medical Services. Data were collected from March 2023 to November 2023, covering records from 2012 to 2023. The obtained data include HC measurements, sex, and medical history. Moreover, in order to accurately calculate each participant’s exact age at the time of measurement, both the date of birth and date of measurement were recorded. This study adhered to the ethical guidelines of the Declaration of Helsinki. Ethical approval was obtained from the Ethics Committees of Al-Zaytoonah University of Jordan (REF#2023-2022/12/30) and the Jordanian Ministry of Health (REF#2023/3/22). Health institutions in Jordan adhere to international HC measurement guidelines, including the International Society for the Advancement of Kinanthropometry (ISAK) standards [31].

### 2.1. Head Circumference Measurement

Health institutions in Jordan adhere to international HC measurement guidelines, including the International Society for the Advancement of Kinanthropometry (ISAK) standards [31]. The Jordanian Health Institution guidelines dictate that trained healthcare staff perform HC measurements by wrapping a non-stretchable tape measure around the widest diameter of the head, which is usually 1–2 finger widths above the eyebrows on the forehead, extending to the most prominent part of the back of the head. The tape is repositioned, and the measurements are repeated to record two readings within 0.2 cm. The average of the two closest measurements are then recorded [12].

### 2.2. Data Selection

Participants were screened for any health conditions. In accordance with the guidelines, only data from healthy infants—those not suffering from any illness or taking medications that could alter the normal pattern of growth—were included.

Data cleaning was performed to enhance quality, refine the dataset, verify accuracy and consistency, and remove duplicates and outliers. Outliers were identified by applying z-scores for HC-for-age. In line with international guidelines, outliers were defined using flags for HC measurements. Z-scores were calculated for each infant based on the study data and international equations. Any z-score exceeding +5 or below −5 was classified as an outlier and was excluded from the dataset [32].

### 2.3. Statistical Analysis

The Statistical Package for the Social Sciences (SPSS), Version 26.0, and RStudio software, Version 4.3.1, were used for statistical analysis [33,34]. All infant readings were converted to standard units to maintain uniformity and consistency: HC was converted to centimeters (cm), and age was computed in days and years. To enable age and gender-based analysis, the data were divided into consecutive age intervals of 120-day increments and were further subcategorized by gender. From birth to 5 years, HC-for-age for Jordanian infants was compared to international standards. Percentiles were calculated based on WHO equations using RStudio’s ‘anthro’ package.

The Box–Cox Power Exponential (BCPE) distribution is defined by four key parameters: μ (location or median), σ (scale or approximate coefficient of variation), ν (skewness, adjusting for symmetry), and τ (kurtosis, describing the power exponential parameter). This distribution underpins the Lambda–Mu–Sigma (LMS) method, where λ (lambda) represents skewness, μ (mu) corresponds to the median, and σ (sigma) describes the coefficient of variation. The LMS method facilitates the development of growth charts and percentile curves that effectively capture developmental changes in children and assess various aspects of growth [35]. Importantly, this method is highly flexible, accommodating a wide range of distributional shapes, including skewed, exponential, and normal (Gaussian) distributions, without imposing a fixed distribution type. Smoothing splines are incorporated to improve the model’s accuracy and alignment with observed data.

Spline smoothing techniques were used during the model selection process to estimate parameters such as skewness, median, sigma, and kurtosis. The choice of smoothing methods prioritized their ability to represent the underlying growth patterns while minimizing overfitting [36]. Statistical measures such as the Akaike Information Criterion (AIC), goodness-of-fit metrics (GD), and degrees of freedom (df) were applied to compare models and identify the best fit for the Jordanian dataset.

Percentile values were employed to evaluate the model’s performance and its ability to capture variability across different levels. Specific percentiles, such as the 3rd, 15th, 50th, 85th, and 97th, were examined to assess how well the model predicted these critical points in the dataset. The inclusion of percentiles in the evaluation process provided further insights into the model’s robustness, ensuring that variability at different levels was accurately captured and represented.

## 3. Results

**Figure 1:**
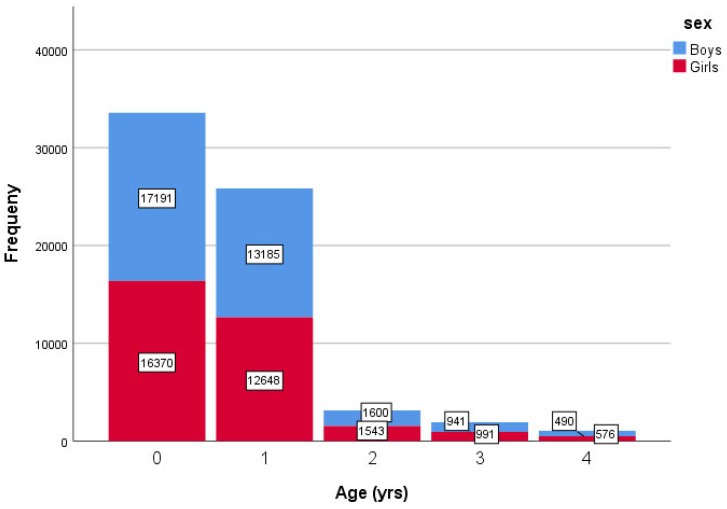
Clustered bar chart showing the distribution of age intervals (in years) by sex.

The analyzed dataset contained 628,456 measurements, comprising 322,952 (51.4%) boys and 305,504 (48.6%) girls. These measurements were taken from 250,276 children, of whom 129,067 (51.6%) were boys and 121,209 (48.4%) were girls, aged 0–1800 days, who met the eligibility criteria for this study. The primary variables collected were age (in days and years) and HC (in centimeters). Figure 1 shows the clustered bar count of age intervals by years and sex.

**Figure 2:**
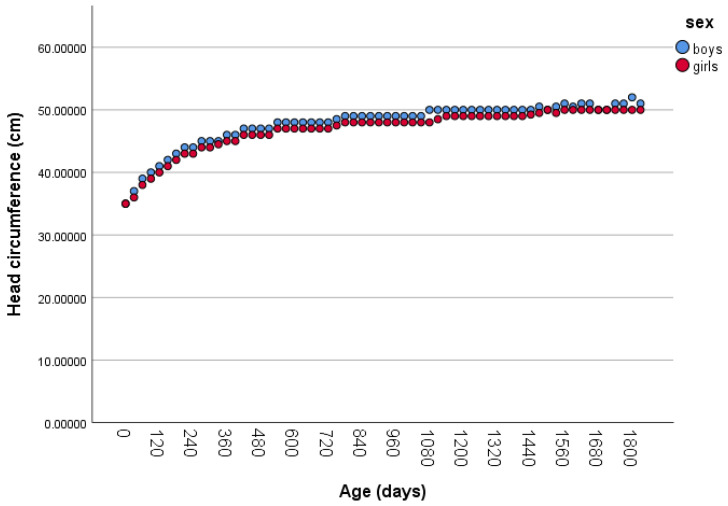
Mean HC growth chart from birth to 1800 days.

Figure 2 illustrates the age-related distribution of mean HC. HC measurements for boys were significantly higher than those for girls, as confirmed using a Mann–Whitney U test (*p* < 0.001).

### 3.1. Growth Chart Models for Jordanian Boys and Girls Aged 0–1800 Days

**Figure 3:**
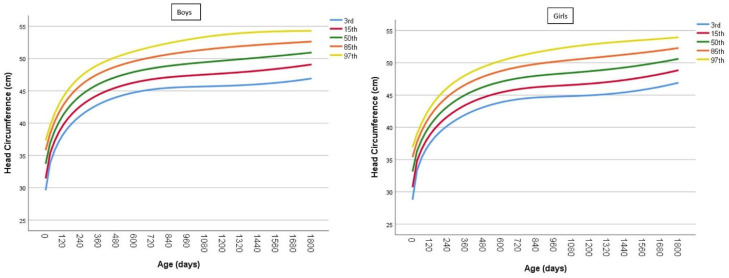
Head circumference (cm) chart for Jordanian (**left**) boys and (**right**) girls, showing the 3rd, 15th, 50th, 85th, and 97th percentiles across different ages.

HC-for-age models were developed for Jordanian boys (Figure 3 left) and girls (Figure 3 right), referencing the 3rd, 15th, 50th, 85th, and 97th percentiles. The most appropriate parameters (μ, σ, ν, and τ) and age-specific splines for the data were determined. The resulting HC-for-age parameters and percentiles for boys and girls are presented in Tables S1 and S2 in the Supplementary Material, with corresponding values for each age.

### 3.2. Comparison of HC in Jordanian Infants and Young Children with WHO Standards

**Figure 4:**
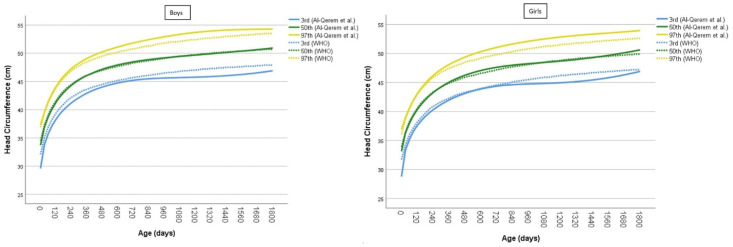
Comparison of the 3rd, 50th, and 97th HC percentiles for Jordanian (**left**) boys and (**right**) girls with WHO reference data.

The percentiles developed by Al-Qerem et al. for Jordanian children were superimposed on the WHO percentiles. For Jordanian boys, the results show that Al-Qerem’s 3rd percentile was consistently below the WHO 3rd percentile curve, while the median percentiles for both models were identical. Al-Qerem’s 97th percentile began to diverge from the WHO 97th percentile curve after 240 days, exceeding the WHO’s values (see Figure 4 left).

For Jordanian girls, Al-Qerem’s 3rd percentile began to deviate significantly below the corresponding WHO 3rd percentile after 840 days. Similarly to boys, the median percentiles were identical for both models, while Al-Qerem’s 97th percentile exceeded the corresponding WHO percentile after 240 days (see Figure 4 right).

## 4. Discussion

The present study aimed to evaluate HC growth patterns in Jordanian infants and children up to five years old and to compare them with established international WHO standards. This study is the first to develop and validate national HC growth charts for children from birth to five years of age. This research provides a comprehensive overview of growth patterns in the Jordanian pediatric population. The findings contribute to understanding the applicability of international growth standards to Jordanian children and highlight the potential benefits of implementing region-specific growth charts.

The results reveal significant differences in HC growth patterns for Jordanian boys and girls compared to WHO standards. Specifically, Al-Qerem’s 3rd percentile for both boys and girls was consistently below the WHO’s 3rd percentile. For boys, this deviation was evident from birth through five years of age, while for girls, it became more noticeable after 840 days. The median percentiles for both the Jordanian and WHO models were similar, suggesting that the average HC growth in Jordanian children closely aligns with international standards. However, the 97th percentile for both boys and girls in Al-Qerem’s model diverged from the WHO’s 97th percentile after 240 days, with Jordanian children displaying larger HC measurements in the upper percentiles.

The similarities in the median percentiles suggest that, on average, Jordanian infants and children experience HC growth comparable to WHO standards under ideal conditions. However, the observed deviations at the lower and upper extremes of the distribution highlight the importance of considering regional variations in growth patterns. For example, the lower 3rd percentile in Jordanian children may reflect genetic, environmental, or socio-economic factors distinct from the populations included in the WHO Multicenter Growth Reference Study [11]. Conversely, the larger HC measurements in the 97th percentile could indicate a higher prevalence of familial macrocephaly or other genetic factors specific to the Jordanian population. On the other hand, a recent review evaluated the limitations of the WHO 2006 growth standards in accurately identifying population-specific HC variations. Median HC measurements in most populations, except for Indian and some Asian neonates, are larger than those reported in the WHO Multicenter Growth Reference Study, aligning with global norms [37]. Therefore, the measurement deviations from the WHO model observed in Al-Qerem’s model for Jordanian infants and children up to five years old may not necessarily represent true estimates, but could instead be attributed to the overestimation of macrocephaly based on WHO growth standards. These standards may not accurately reflect the genetic, environmental, or socio-economic factors unique to the Jordanian population [14], potentially leading to the misclassification of larger head circumferences as macrocephalic.

The necessity of region-specific growth charts is particularly evident in clinical practice [38], where they play a critical role in identifying and managing conditions such as microcephaly and macrocephaly. For instance, underestimating HC in Jordanian children at the lower end of the distribution could delay the diagnosis of microcephaly, while overestimating HC at the upper end may lead to unnecessary investigations for macrocephaly. Therefore, developing national growth charts tailored to the specific growth patterns of Jordanian children, as achieved in this study, represents a crucial advancement in enhancing pediatric healthcare and ensuring more precise assessments in the region.

## 5. Strengths and Limitations

This study included a large sample size, which strengthened the robustness and reliability of the data analysis, providing greater statistical power and more precise estimates of HC growth patterns among infants and children up to five years of age. Furthermore, the use of the advanced LMS statistical method ensured the development of the most appropriate models for the data.

However, this study has several limitations. Although the sample size was substantial, it was limited to children enrolled in the Hakeem database, potentially excluding those outside the formal healthcare system (i.e., Public, military, and University hospitals); however, this system serves 86% of the Jordanian population [39]. Additionally, the lack of consideration for socioeconomic data and material characteristics limited the ability to evaluate their impact on HC measurements and growth patterns [40,41]. Moreover, although the Jordanian Ministry of Health adheres to international guidelines for HC measurements, the accuracy of the measurements was not independently verified by the authors of the present study. Finally, due to the common focus on HC measurements for younger children, the data available for older children were significantly fewer than those for younger ages.

## 6. Conclusions

This study offers important insights into HC growth patterns among Jordanian children, emphasizing the need for region-specific growth standards. The development of Al-Qerem’s HC-for-age percentiles represents a significant advancement, providing clinicians and public health professionals in Jordan with a valuable tool for the accurate assessment and monitoring of children’s growth. While international standards remain valuable as a reference, this research highlights the importance of adapting these benchmarks to reflect local contexts, thereby helping to ensure better health outcomes for children in Jordan.

## Data Availability

The dataset supporting the conclusions of this article is available in the Zenodo repository: https://doi.org/10.5281/zenodo.14672462.

## Supplementary Materials

The following supporting information can be downloaded at: https://www.mdpi.com/article/10.3390/children12020224/s1, Table S1: Centiles and equation parameters for HC-for-age in Jordanian boys aged 0–5 years; Table S2: Centiles and equation parameters for HC-for-age in Jordanian girls aged 0–5 years.
