# Supplementary material for: The Development of Standardized National Head Circumference Growth Charts for Jordanian Children Aged 0–5 Years: A Longitudinal and Cross-Sectional Study
Source: Children (Basel). 2025 Feb 13;12(2):224. doi: 10.3390/children12020224 (PMC11854488; doi:10.3390/children12020224)
Supplement: Supplementary file 1 [file children-12-00224-s001.zip › children-3457120-supplementary.pdf]

Table S1. Centiles and equation parameters for HC-for-age in Jordanian boys aged 0–5 years

Age (days)	M	S	Tau	Nu	C1	C3	C5	C10	C15	C25	C50	C75	C85	C90	C95	C97	C99
0	33.70	-2.79	0.81	2.12	28.6	29.6	30.1	30.9	31.4	32.2	33.7	35.1	35.8	36.3	36.9	37.3	38.1
30	36.89	-3.20	0.57	2.60	33.0	33.8	34.3	34.9	35.3	35.9	36.9	37.8	38.4	38.7	39.2	39.6	40.2
60	38.74	-3.26	0.57	2.45	34.9	35.8	36.2	36.8	37.2	37.8	38.7	39.7	40.2	40.6	41.1	41.4	42.1
90	40.17	-3.29	0.57	2.22	36.4	37.2	37.6	38.2	38.6	39.2	40.2	41.1	41.6	42.0	42.5	42.9	43.6
120	41.32	-3.31	0.56	2.06	37.5	38.3	38.7	39.4	39.8	40.3	41.3	42.3	42.8	43.2	43.7	44.1	44.8
150	42.27	-3.32	0.56	1.95	38.4	39.2	39.7	40.3	40.7	41.3	42.3	43.2	43.8	44.2	44.7	45.1	45.8
180	43.07	-3.32	0.56	1.87	39.2	40.0	40.4	41.1	41.5	42.1	43.1	44.1	44.6	45.0	45.6	45.9	46.7
210	43.75	-3.33	0.55	1.82	39.8	40.7	41.1	41.7	42.1	42.7	43.7	44.7	45.3	45.7	46.3	46.7	47.4
240	44.34	-3.33	0.55	1.76	40.4	41.2	41.7	42.3	42.7	43.3	44.3	45.3	45.9	46.3	46.9	47.3	48.0
270	44.85	-3.34	0.55	1.71	40.9	41.7	42.2	42.8	43.2	43.8	44.9	45.9	46.4	46.8	47.4	47.8	48.6
300	45.30	-3.34	0.54	1.66	41.3	42.2	42.6	43.3	43.7	44.3	45.3	46.3	46.9	47.3	47.9	48.3	49.1
330	45.70	-3.35	0.54	1.61	41.7	42.6	43.0	43.7	44.1	44.7	45.7	46.7	47.3	47.7	48.3	48.7	49.5
360	46.06	-3.36	0.53	1.55	42.1	42.9	43.4	44.0	44.4	45.0	46.1	47.1	47.7	48.1	48.7	49.1	49.9
390	46.38	-3.36	0.53	1.49	42.4	43.2	43.7	44.3	44.8	45.3	46.4	47.4	48.0	48.4	49.0	49.4	50.2
420	46.67	-3.36	0.52	1.43	42.7	43.5	44.0	44.6	45.0	45.6	46.7	47.7	48.3	48.7	49.3	49.7	50.5
450	46.93	-3.37	0.52	1.37	42.9	43.8	44.2	44.9	45.3	45.9	46.9	48.0	48.5	49.0	49.6	50.0	50.8
480	47.17	-3.37	0.51	1.30	43.2	44.0	44.5	45.1	45.5	46.1	47.2	48.2	48.8	49.2	49.8	50.3	51.1
510	47.39	-3.37	0.51	1.23	43.4	44.2	44.7	45.3	45.8	46.4	47.4	48.4	49.0	49.4	50.1	50.5	51.3
540	47.59	-3.37	0.51	1.15	43.6	44.4	44.9	45.5	45.9	46.5	47.6	48.6	49.2	49.6	50.3	50.7	51.6
570	47.77	-3.37	0.50	1.07	43.7	44.6	45.0	45.7	46.1	46.7	47.8	48.8	49.4	49.8	50.5	50.9	51.8
600	47.94	-3.36	0.50	0.99	43.9	44.8	45.2	45.9	46.3	46.9	47.9	49.0	49.6	50.0	50.7	51.1	52.0
630	48.09	-3.36	0.49	0.91	44.0	44.9	45.3	46.0	46.4	47.0	48.1	49.1	49.8	50.2	50.9	51.3	52.2
660	48.23	-3.35	0.49	0.83	44.1	45.0	45.5	46.1	46.6	47.2	48.2	49.3	49.9	50.3	51.0	51.5	52.4
690	48.36	-3.35	0.48	0.76	44.2	45.1	45.6	46.2	46.7	47.3	48.4	49.4	50.1	50.5	51.2	51.7	52.6

720	48.48	-3.34	0.48	0.69	44.3	45.2	45.7	46.3	46.8	47.4	48.5	49.6	50.2	50.6	51.3	51.8	52.8
750	48.59	-3.33	0.47	0.63	44.4	45.3	45.8	46.4	46.9	47.5	48.6	49.7	50.3	50.8	51.5	52.0	52.9
780	48.70	-3.32	0.47	0.57	44.5	45.4	45.8	46.5	47.0	47.6	48.7	49.8	50.4	50.9	51.6	52.1	53.1
810	48.79	-3.31	0.46	0.53	44.5	45.4	45.9	46.6	47.1	47.7	48.8	49.9	50.6	51.0	51.8	52.3	53.3
840	48.88	-3.30	0.46	0.49	44.5	45.5	46.0	46.7	47.1	47.8	48.9	50.0	50.7	51.2	51.9	52.4	53.4
870	48.96	-3.29	0.45	0.45	44.6	45.5	46.0	46.7	47.2	47.8	49.0	50.1	50.8	51.3	52.0	52.5	53.6
900	49.04	-3.28	0.45	0.43	44.6	45.5	46.0	46.8	47.2	47.9	49.0	50.2	50.9	51.4	52.1	52.7	53.7
930	49.11	-3.27	0.44	0.42	44.6	45.6	46.1	46.8	47.3	48.0	49.1	50.3	51.0	51.5	52.3	52.8	53.9
960	49.18	-3.26	0.44	0.41	44.6	45.6	46.1	46.9	47.3	48.0	49.2	50.4	51.1	51.6	52.4	52.9	54.0
990	49.25	-3.24	0.43	0.41	44.6	45.6	46.1	46.9	47.4	48.1	49.2	50.4	51.2	51.7	52.5	53.0	54.1
1020	49.31	-3.23	0.43	0.43	44.6	45.6	46.2	46.9	47.4	48.1	49.3	50.5	51.2	51.8	52.6	53.1	54.3
1050	49.38	-3.22	0.42	0.45	44.6	45.7	46.2	47.0	47.5	48.2	49.4	50.6	51.3	51.8	52.7	53.2	54.4
1080	49.44	-3.22	0.42	0.48	44.6	45.7	46.2	47.0	47.5	48.2	49.4	50.7	51.4	51.9	52.8	53.3	54.5
1110	49.50	-3.21	0.42	0.52	44.6	45.7	46.2	47.0	47.6	48.3	49.5	50.7	51.5	52.0	52.9	53.4	54.6
1140	49.55	-3.20	0.41	0.56	44.6	45.7	46.3	47.1	47.6	48.3	49.6	50.8	51.5	52.1	52.9	53.5	54.7
1170	49.61	-3.19	0.41	0.62	44.6	45.7	46.3	47.1	47.6	48.4	49.6	50.9	51.6	52.2	53.0	53.6	54.8
1200	49.67	-3.19	0.40	0.68	44.6	45.8	46.3	47.2	47.7	48.4	49.7	50.9	51.7	52.2	53.1	53.7	54.9
1230	49.73	-3.18	0.40	0.76	44.6	45.8	46.4	47.2	47.7	48.5	49.7	51.0	51.7	52.3	53.2	53.8	54.9
1260	49.78	-3.18	0.39	0.84	44.6	45.8	46.4	47.2	47.8	48.5	49.8	51.0	51.8	52.3	53.2	53.8	55.0
1290	49.84	-3.18	0.39	0.92	44.7	45.8	46.4	47.3	47.8	48.6	49.8	51.1	51.9	52.4	53.3	53.9	55.1
1320	49.90	-3.18	0.38	1.02	44.7	45.9	46.5	47.3	47.9	48.6	49.9	51.1	51.9	52.5	53.3	53.9	55.1
1350	49.96	-3.18	0.38	1.12	44.7	45.9	46.5	47.4	47.9	48.7	50.0	51.2	52.0	52.5	53.4	54.0	55.2
1380	50.02	-3.18	0.37	1.23	44.7	45.9	46.5	47.4	48.0	48.8	50.0	51.3	52.0	52.6	53.4	54.0	55.2
1410	50.07	-3.18	0.37	1.35	44.7	46.0	46.6	47.5	48.1	48.8	50.1	51.3	52.1	52.6	53.5	54.1	55.2
1440	50.13	-3.18	0.36	1.48	44.8	46.0	46.6	47.5	48.1	48.9	50.1	51.4	52.1	52.7	53.5	54.1	55.3
1470	50.20	-3.19	0.36	1.61	44.8	46.1	46.7	47.6	48.2	49.0	50.2	51.4	52.2	52.7	53.5	54.1	55.3
1500	50.26	-3.19	0.35	1.75	44.8	46.1	46.8	47.7	48.2	49.0	50.3	51.5	52.2	52.7	53.6	54.2	55.3

1530	50.32	-3.20	0.35	1.89	44.9	46.2	46.8	47.7	48.3	49.1	50.3	51.5	52.3	52.8	53.6	54.2	55.3
1560	50.38	-3.21	0.34	2.04	44.9	46.2	46.9	47.8	48.4	49.2	50.4	51.6	52.3	52.8	53.6	54.2	55.3
1590	50.45	-3.22	0.34	2.20	45.0	46.3	47.0	47.9	48.5	49.2	50.4	51.6	52.3	52.9	53.7	54.2	55.3
1620	50.51	-3.23	0.33	2.37	45.0	46.4	47.0	48.0	48.5	49.3	50.5	51.7	52.4	52.9	53.7	54.2	55.3
1650	50.58	-3.24	0.33	2.54	45.1	46.5	47.1	48.1	48.6	49.4	50.6	51.7	52.4	52.9	53.7	54.2	55.3
1680	50.65	-3.25	0.32	2.72	45.1	46.5	47.2	48.1	48.7	49.5	50.6	51.8	52.5	53.0	53.7	54.3	55.3
1710	50.72	-3.26	0.32	2.90	45.2	46.6	47.3	48.2	48.8	49.6	50.7	51.8	52.5	53.0	53.7	54.3	55.3
1740	50.79	-3.28	0.32	3.09	45.3	46.7	47.4	48.3	48.9	49.6	50.8	51.9	52.5	53.0	53.8	54.3	55.3
1770	50.86	-3.29	0.31	3.28	45.4	46.8	47.5	48.4	49.0	49.7	50.9	51.9	52.6	53.1	53.8	54.3	55.2
1800	50.93	-3.31	0.31	3.48	45.5	46.9	47.6	48.5	49.1	49.8	50.9	52.0	52.6	53.1	53.8	54.3	55.2

Table S2. Centiles and equation parameters for HC-for-age in Jordanian girls aged 0-5 years

Age (days)	M	S	Tau	Nu	C1	C3	C5	C10	C15	C25	C50	C75	C85	C90	C95	C97	C99
0	33.14	-2.72	0.86	2.16	27.8	28.8	29.3	30.1	30.7	31.6	33.1	34.6	35.4	35.9	36.5	36.9	37.7
30	36.31	-3.22	0.52	2.36	32.5	33.4	33.8	34.4	34.8	35.4	36.3	37.2	37.7	38.1	38.6	39.0	39.6
60	37.98	-3.30	0.55	1.77	34.5	35.2	35.6	36.2	36.5	37.1	38.0	38.9	39.4	39.7	40.2	40.6	41.3
90	39.29	-3.33	0.56	1.34	35.9	36.6	36.9	37.5	37.9	38.4	39.3	40.2	40.7	41.1	41.6	42.0	42.6
120	40.36	-3.34	0.55	1.11	36.9	37.6	38.0	38.5	38.9	39.4	40.4	41.3	41.8	42.2	42.7	43.1	43.8
150	41.26	-3.34	0.54	1.03	37.7	38.5	38.8	39.4	39.8	40.3	41.3	42.2	42.7	43.1	43.7	44.1	44.8
180	42.03	-3.34	0.52	1.03	38.4	39.2	39.6	40.1	40.5	41.1	42.0	43.0	43.5	43.9	44.5	44.9	45.6
210	42.69	-3.34	0.51	1.05	39.0	39.8	40.2	40.8	41.2	41.7	42.7	43.7	44.2	44.6	45.2	45.6	46.4
240	43.28	-3.34	0.50	1.09	39.5	40.3	40.7	41.3	41.7	42.3	43.3	44.2	44.8	45.2	45.8	46.2	47.0
270	43.80	-3.34	0.50	1.12	40.0	40.8	41.2	41.8	42.2	42.8	43.8	44.8	45.3	45.7	46.4	46.8	47.6
300	44.26	-3.34	0.49	1.15	40.4	41.2	41.7	42.3	42.7	43.3	44.3	45.3	45.8	46.2	46.9	47.3	48.1
330	44.68	-3.34	0.49	1.16	40.8	41.6	42.1	42.7	43.1	43.7	44.7	45.7	46.3	46.7	47.3	47.7	48.5
360	45.06	-3.34	0.49	1.15	41.1	42.0	42.4	43.1	43.5	44.1	45.1	46.1	46.6	47.0	47.7	48.1	48.9
390	45.40	-3.34	0.49	1.14	41.5	42.3	42.7	43.4	43.8	44.4	45.4	46.4	47.0	47.4	48.0	48.5	49.3
420	45.71	-3.35	0.48	1.11	41.8	42.6	43.0	43.7	44.1	44.7	45.7	46.7	47.3	47.7	48.4	48.8	49.6
450	46.00	-3.35	0.48	1.07	42.0	42.9	43.3	44.0	44.4	45.0	46.0	47.0	47.6	48.0	48.7	49.1	49.9
480	46.25	-3.35	0.48	1.01	42.3	43.1	43.6	44.2	44.6	45.2	46.3	47.3	47.9	48.3	48.9	49.4	50.2
510	46.49	-3.35	0.48	0.95	42.5	43.3	43.8	44.4	44.9	45.5	46.5	47.5	48.1	48.5	49.2	49.6	50.5
540	46.70	-3.34	0.48	0.87	42.7	43.5	44.0	44.6	45.1	45.7	46.7	47.7	48.3	48.8	49.4	49.9	50.8
570	46.89	-3.34	0.48	0.78	42.9	43.7	44.2	44.8	45.3	45.9	46.9	47.9	48.5	49.0	49.7	50.1	51.0
600	47.07	-3.34	0.48	0.68	43.0	43.9	44.3	45.0	45.4	46.0	47.1	48.1	48.7	49.2	49.9	50.3	51.2
630	47.23	-3.33	0.48	0.58	43.2	44.0	44.5	45.1	45.6	46.2	47.2	48.3	48.9	49.4	50.0	50.5	51.4
660	47.37	-3.33	0.48	0.47	43.3	44.2	44.6	45.3	45.7	46.3	47.4	48.4	49.1	49.5	50.2	50.7	51.6
690	47.50	-3.32	0.47	0.36	43.4	44.3	44.7	45.4	45.8	46.4	47.5	48.6	49.2	49.7	50.4	50.9	51.8

720	47.62	-3.31	0.47	0.26	43.5	44.4	44.8	45.5	45.9	46.5	47.6	48.7	49.4	49.8	50.6	51.0	52.0
750	47.73	-3.30	0.47	0.16	43.6	44.5	44.9	45.6	46.0	46.6	47.7	48.8	49.5	50.0	50.7	51.2	52.2
780	47.82	-3.29	0.47	0.07	43.6	44.5	45.0	45.7	46.1	46.7	47.8	48.9	49.6	50.1	50.8	51.4	52.4
810	47.91	-3.28	0.46	-0.02	43.7	44.6	45.0	45.7	46.2	46.8	47.9	49.0	49.7	50.2	51.0	51.5	52.5
840	47.99	-3.27	0.46	-0.09	43.7	44.6	45.1	45.8	46.2	46.9	48.0	49.1	49.8	50.3	51.1	51.6	52.7
870	48.06	-3.26	0.46	-0.16	43.8	44.7	45.1	45.8	46.3	46.9	48.1	49.2	49.9	50.4	51.2	51.8	52.8
900	48.12	-3.25	0.45	-0.22	43.8	44.7	45.2	45.9	46.3	47.0	48.1	49.3	50.0	50.5	51.3	51.9	53.0
930	48.18	-3.24	0.45	-0.27	43.8	44.7	45.2	45.9	46.4	47.0	48.2	49.4	50.1	50.6	51.4	52.0	53.1
960	48.24	-3.23	0.45	-0.31	43.8	44.7	45.2	45.9	46.4	47.1	48.2	49.4	50.2	50.7	51.5	52.1	53.3
990	48.30	-3.22	0.44	-0.34	43.8	44.8	45.2	46.0	46.5	47.1	48.3	49.5	50.2	50.8	51.6	52.2	53.4
1020	48.35	-3.21	0.44	-0.36	43.8	44.8	45.3	46.0	46.5	47.2	48.3	49.6	50.3	50.9	51.7	52.3	53.5
1050	48.40	-3.21	0.43	-0.37	43.9	44.8	45.3	46.0	46.5	47.2	48.4	49.6	50.4	50.9	51.8	52.4	53.6
1080	48.46	-3.20	0.43	-0.37	43.9	44.8	45.3	46.1	46.6	47.3	48.5	49.7	50.5	51.0	51.9	52.5	53.7
1110	48.51	-3.19	0.43	-0.36	43.9	44.9	45.4	46.1	46.6	47.3	48.5	49.8	50.5	51.1	52.0	52.6	53.8
1140	48.57	-3.19	0.42	-0.33	43.9	44.9	45.4	46.2	46.7	47.4	48.6	49.8	50.6	51.2	52.1	52.7	53.9
1170	48.63	-3.18	0.42	-0.30	43.9	44.9	45.4	46.2	46.7	47.4	48.6	49.9	50.7	51.2	52.1	52.8	54.0
1200	48.69	-3.18	0.41	-0.26	43.9	45.0	45.5	46.3	46.8	47.5	48.7	49.9	50.7	51.3	52.2	52.8	54.1
1230	48.76	-3.17	0.41	-0.21	44.0	45.0	45.5	46.3	46.8	47.5	48.8	50.0	50.8	51.4	52.3	52.9	54.2
1260	48.82	-3.17	0.40	-0.15	44.0	45.0	45.6	46.4	46.9	47.6	48.8	50.1	50.9	51.4	52.3	53.0	54.3
1290	48.89	-3.17	0.40	-0.08	44.0	45.1	45.6	46.4	46.9	47.7	48.9	50.1	50.9	51.5	52.4	53.0	54.3
1320	48.96	-3.17	0.40	0.01	44.1	45.1	45.7	46.5	47.0	47.7	49.0	50.2	51.0	51.6	52.5	53.1	54.4
1350	49.04	-3.18	0.39	0.10	44.1	45.2	45.8	46.6	47.1	47.8	49.0	50.3	51.1	51.6	52.5	53.2	54.4
1380	49.12	-3.18	0.39	0.20	44.2	45.3	45.8	46.6	47.2	47.9	49.1	50.4	51.1	51.7	52.6	53.2	54.5
1410	49.20	-3.18	0.38	0.31	44.3	45.3	45.9	46.7	47.3	48.0	49.2	50.4	51.2	51.8	52.7	53.3	54.5
1440	49.29	-3.19	0.38	0.42	44.3	45.4	46.0	46.8	47.3	48.1	49.3	50.5	51.3	51.8	52.7	53.3	54.6
1470	49.37	-3.19	0.37	0.55	44.4	45.5	46.1	46.9	47.4	48.2	49.4	50.6	51.3	51.9	52.8	53.4	54.6
1500	49.47	-3.20	0.37	0.69	44.5	45.6	46.2	47.0	47.5	48.3	49.5	50.7	51.4	52.0	52.8	53.4	54.6

1530	49.56	-3.21	0.37	0.83	44.6	45.7	46.3	47.1	47.6	48.4	49.6	50.8	51.5	52.0	52.9	53.5	54.7
1560	49.67	-3.22	0.36	0.98	44.7	45.8	46.4	47.2	47.8	48.5	49.7	50.8	51.6	52.1	52.9	53.5	54.7
1590	49.77	-3.23	0.36	1.14	44.8	45.9	46.5	47.3	47.9	48.6	49.8	50.9	51.7	52.2	53.0	53.6	54.7
1620	49.88	-3.24	0.36	1.31	44.9	46.0	46.6	47.5	48.0	48.7	49.9	51.0	51.7	52.3	53.1	53.6	54.7
1650	49.99	-3.26	0.35	1.49	45.0	46.2	46.8	47.6	48.1	48.8	50.0	51.1	51.8	52.3	53.1	53.7	54.8
1680	50.11	-3.27	0.35	1.67	45.1	46.3	46.9	47.7	48.3	49.0	50.1	51.2	51.9	52.4	53.2	53.7	54.8
1710	50.23	-3.29	0.34	1.86	45.2	46.4	47.0	47.9	48.4	49.1	50.2	51.3	52.0	52.5	53.3	53.8	54.8
1740	50.35	-3.30	0.34	2.06	45.4	46.6	47.2	48.0	48.5	49.2	50.4	51.4	52.1	52.6	53.3	53.8	54.8
1770	50.48	-3.32	0.34	2.27	45.5	46.8	47.3	48.2	48.7	49.4	50.5	51.5	52.2	52.7	53.4	53.9	54.9
1800	50.61	-3.34	0.33	2.48	45.7	46.9	47.5	48.3	48.9	49.5	50.6	51.7	52.3	52.7	53.5	53.9	54.9
